# 2,4-Dichloro­benzaldehyde 4-methyl­thio­semicarbazone

**DOI:** 10.1107/S1600536810049743

**Published:** 2010-12-04

**Authors:** Rongchun Li

**Affiliations:** a Department of Chemistry, Dezhou University, Dezhou 253023, People’s Republic of China

## Abstract

The mol­ecule of the title compound, C_9_H_9_Cl_2_N_3_S, has an *E* configuration about the C=N bond. In the crystal, mol­ecules are linked through inter­molecular N—H⋯S hydrogen bonds, forming zigzag chains along the *a* axis.

## Related literature

For background to Schiff bases derived from thio­semicarbazone and its derivatives, see: Casas *et al.* (2001 ▶); Beraldo *et al.* (2001 ▶); Jouad *et al.* (2002 ▶); Swearingen *et al.* (2002 ▶). For a similar structure reported recently by the author, see: Li (2010 ▶). For bond-length data, see: Allen *et al.* (1987 ▶). For similar structures, see: Selvanayagam *et al.* (2002 ▶); Karakurt *et al.* (2003 ▶); Bernhardt *et al.* (2003 ▶); Sampath *et al.* (2003 ▶).
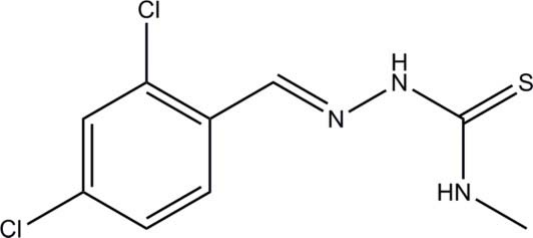


## Experimental

### 

#### Crystal data

C_9_H_9_Cl_2_N_3_S
*M* *_r_* = 262.15
Monoclinic, 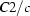

*a* = 13.444 (3) Å
*b* = 9.3299 (19) Å
*c* = 18.499 (4) Å
β = 92.160 (2)°
*V* = 2318.7 (8) Å^3^
*Z* = 8
Mo *K*α radiation
μ = 0.71 mm^−1^
*T* = 298 K
0.18 × 0.17 × 0.13 mm

#### Data collection

Bruker APEXII CCD area-detector diffractometer
Absorption correction: multi-scan (*SADABS*; Sheldrick, 2004 ▶) *T*_min_ = 0.883, *T*_max_ = 0.913
7167 measured reflections
2518 independent reflections
1956 reflections with *I* > 2σ(*I*)
*R*_int_ = 0.029

#### Refinement

*R*[*F* ^2^ > 2σ(*F* ^2^)] = 0.037
*wR*(*F* ^2^) = 0.092
*S* = 1.05
2518 reflections
143 parameters
2 restraints
H atoms treated by a mixture of independent and constrained refinement
Δρ_max_ = 0.23 e Å^−3^
Δρ_min_ = −0.35 e Å^−3^

### 

Data collection: *APEX2* (Bruker, 2004 ▶); cell refinement: *SAINT* (Bruker, 2004 ▶); data reduction: *SAINT*; program(s) used to solve structure: *SHELXS97* (Sheldrick, 2008 ▶); program(s) used to refine structure: *SHELXL97* (Sheldrick, 2008 ▶); molecular graphics: *SHELXTL* (Sheldrick, 2008 ▶); software used to prepare material for publication: *SHELXTL*.

## Supplementary Material

Crystal structure: contains datablocks global, I. DOI: 10.1107/S1600536810049743/hg2763sup1.cif

Structure factors: contains datablocks I. DOI: 10.1107/S1600536810049743/hg2763Isup2.hkl

Additional supplementary materials:  crystallographic information; 3D view; checkCIF report

## Figures and Tables

**Table 1:** Hydrogen-bond geometry (Å, °)

*D*—H⋯*A*	*D*—H	H⋯*A*	*D*⋯*A*	*D*—H⋯*A*
N2—H2⋯S1^i^	0.90 (1)	2.54 (1)	3.4169 (18)	167 (2)
N3—H3⋯S1^ii^	0.89 (1)	2.77 (2)	3.491 (2)	139 (2)

Symmetry codes: (i) 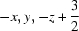; (ii) 
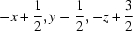
.

**Fig. 1.**
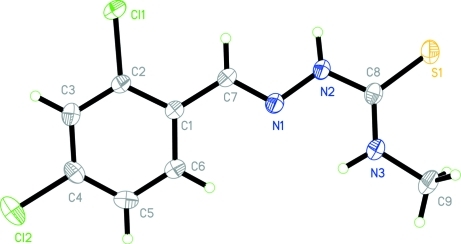
The molecular structure of the title compound, showing 30% probability displacement ellipsoids and the atom-numbering scheme.

**Fig. 2.**
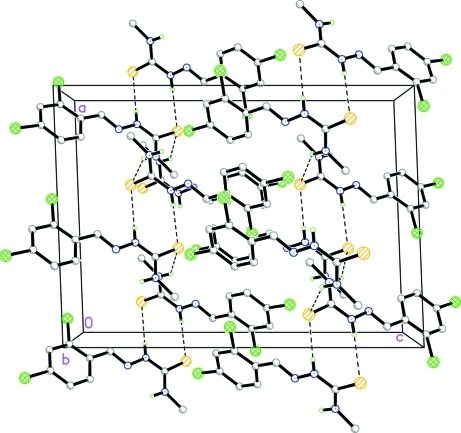
The crystal packing of the title compound, viewed along the b axis.

## supplementary crystallographic information

### Comment

Thiosemicarbazone and its derivatives are important materials for the preparation of Schiff bases (Casas *et al.*, 2001; Beraldo *et al.*, 2001; Jouad *et al.*, 2002; Swearingen *et al.*, 2002). As a continuation of the work on the structures of such compounds (Li, 2010), in this paper, the title new Schiff base compound derived from the condensation of 2,4-dichlorobenzaldehyde with 4-methylthiosemicarbazone is reported.

The molecule of the title compound, Fig. 1, possesses an *E* configuration about the C7═N1 bond. The bond lengths have normal values (Allen *et al.*, 1987), and are comparable to those observed in similar compounds (Selvanayagam *et al.*, 2002; Karakurt *et al.*, 2003; Bernhardt *et al.*, 2003; Sampath *et al.*, 2003).

In the crystal, molecules are linked through intermolecular N—H···S hydrogen bonds (Table 1), to form zigzag chains along the *a* axis (Fig. 2).

### Experimental

The title compound was prepared by the Schiff base condensation of equimolar quantities of 2,4-dichlorobenzaldehyde (0.174 g, 1 mmol) with 4-methylthiosemicarbazone (0.105 g, 1 mmol) in methanol. The excess methanol was removed by distillation. Colourless block shaped single crystals were obatined by slow evaporation of an ethanol solution of the product in air.

### Refinement

The amino H atoms were located in a difference map and refined with N—H distance restrained to 0.90 (1) Å. The remaining H atoms were positioned geometrically (C—H = 0.93–0.96 Å) and refined using a riding model, with *U*_iso_(H) = 1.2*U*_eq_(C) and 1.5*U*_eq_(C9).

### Crystal data

**Table:**

C_9_H_9_Cl_2_N_3_S	*F*(000) = 1072
*M**_r_* = 262.15	*D*_x_ = 1.502 Mg m^−^^3^
Monoclinic, *C*2/*c*	Mo *K*α radiation, λ = 0.71073 Å
Hall symbol: -C 2yc	Cell parameters from 2253 reflections
*a* = 13.444 (3) Å	θ = 2.6–27.3°
*b* = 9.3299 (19) Å	µ = 0.71 mm^−^^1^
*c* = 18.499 (4) Å	*T* = 298 K
β = 92.160 (2)°	Block, colourless
*V* = 2318.7 (8) Å^3^	0.18 × 0.17 × 0.13 mm
*Z* = 8	

### Data collection

**Table:**

Bruker APEXII CCD area-detector diffractometer	2518 independent reflections
Radiation source: fine-focus sealed tube	1956 reflections with *I* > 2σ(*I*)
graphite	*R*_int_ = 0.029
ω scans	θ_max_ = 27.0°, θ_min_ = 2.2°
Absorption correction: multi-scan (*SADABS*; Sheldrick, 2004)	*h* = −16→17
*T*_min_ = 0.883, *T*_max_ = 0.913	*k* = −11→11
7167 measured reflections	*l* = −12→23

### Refinement

**Table:**

Refinement on *F*^2^	Primary atom site location: structure-invariant direct methods
Least-squares matrix: full	Secondary atom site location: difference Fourier map
*R*[*F*^2^ > 2σ(*F*^2^)] = 0.037	Hydrogen site location: inferred from neighbouring sites
*wR*(*F*^2^) = 0.092	H atoms treated by a mixture of independent and constrained refinement
*S* = 1.05	*w* = 1/[σ^2^(*F*_o_^2^) + (0.0407*P*)^2^ + 0.9729*P*] where *P* = (*F*_o_^2^ + 2*F*_c_^2^)/3
2518 reflections	(Δ/σ)_max_ < 0.001
143 parameters	Δρ_max_ = 0.23 e Å^−^^3^
2 restraints	Δρ_min_ = −0.35 e Å^−^^3^

### Special details

**Table:**

Geometry. All e.s.d.'s (except the e.s.d. in the dihedral angle between two l.s. planes) are estimated using the full covariance matrix. The cell e.s.d.'s are taken into account individually in the estimation of e.s.d.'s in distances, angles and torsion angles; correlations between e.s.d.'s in cell parameters are only used when they are defined by crystal symmetry. An approximate (isotropic) treatment of cell e.s.d.'s is used for estimating e.s.d.'s involving l.s. planes.
Refinement. Refinement of *F*^2^ against ALL reflections. The weighted *R*-factor *wR* and goodness of fit *S* are based on *F*^2^, conventional *R*-factors *R* are based on *F*, with *F* set to zero for negative *F*^2^. The threshold expression of *F*^2^ > σ(*F*^2^) is used only for calculating *R*-factors(gt) *etc*. and is not relevant to the choice of reflections for refinement. *R*-factors based on *F*^2^ are statistically about twice as large as those based on *F*, and *R*- factors based on ALL data will be even larger.

### Fractional atomic coordinates and isotropic or equivalent isotropic displacement parameters (Å^2^)

**Table:**

	*x*	*y*	*z*	*U*_iso_*/*U*_eq_	
Cl1	−0.06826 (4)	0.82058 (6)	1.03663 (3)	0.05201 (18)	
Cl2	0.14697 (5)	0.37973 (7)	1.13869 (4)	0.0674 (2)	
N1	0.13058 (12)	0.83265 (18)	0.85768 (9)	0.0383 (4)	
N2	0.10483 (12)	0.93140 (19)	0.80525 (9)	0.0410 (4)	
N3	0.24558 (13)	0.8704 (2)	0.74802 (10)	0.0433 (4)	
S1	0.12967 (4)	1.06871 (6)	0.68333 (3)	0.04588 (17)	
C1	0.09166 (13)	0.7175 (2)	0.96685 (10)	0.0317 (4)	
C2	0.03225 (14)	0.7064 (2)	1.02693 (10)	0.0344 (4)	
C3	0.04938 (16)	0.6053 (2)	1.08024 (11)	0.0409 (5)	
H3A	0.0089	0.6004	1.1198	0.049*	
C4	0.12731 (16)	0.5122 (2)	1.07367 (11)	0.0422 (5)	
C5	0.18905 (16)	0.5198 (2)	1.01613 (12)	0.0452 (5)	
H5	0.2421	0.4566	1.0127	0.054*	
C6	0.17114 (14)	0.6221 (2)	0.96390 (11)	0.0396 (5)	
H6	0.2134	0.6280	0.9253	0.047*	
C7	0.07061 (14)	0.8209 (2)	0.90939 (10)	0.0349 (4)	
H7	0.0139	0.8779	0.9103	0.042*	
C8	0.16428 (14)	0.9497 (2)	0.74839 (11)	0.0360 (5)	
C9	0.32200 (18)	0.8847 (3)	0.69507 (14)	0.0600 (7)	
H9A	0.2927	0.8722	0.6473	0.090*	
H9B	0.3723	0.8131	0.7039	0.090*	
H9C	0.3515	0.9782	0.6990	0.090*	
H2	0.0484 (12)	0.982 (2)	0.8085 (14)	0.080*	
H3	0.258 (2)	0.810 (2)	0.7848 (10)	0.080*	

### Atomic displacement parameters (Å^2^)

**Table:**

	*U*^11^	*U*^22^	*U*^33^	*U*^12^	*U*^13^	*U*^23^
Cl1	0.0503 (3)	0.0534 (3)	0.0537 (4)	0.0147 (3)	0.0206 (3)	0.0003 (3)
Cl2	0.0850 (5)	0.0555 (4)	0.0599 (4)	−0.0016 (3)	−0.0195 (3)	0.0188 (3)
N1	0.0368 (9)	0.0442 (9)	0.0341 (9)	0.0010 (7)	0.0054 (7)	0.0026 (8)
N2	0.0372 (10)	0.0511 (10)	0.0355 (10)	0.0040 (8)	0.0097 (8)	0.0096 (8)
N3	0.0419 (10)	0.0483 (10)	0.0407 (11)	0.0016 (8)	0.0141 (8)	0.0049 (8)
S1	0.0463 (3)	0.0495 (3)	0.0421 (3)	−0.0076 (3)	0.0037 (2)	0.0102 (2)
C1	0.0299 (10)	0.0328 (10)	0.0325 (10)	−0.0020 (8)	0.0020 (8)	−0.0036 (8)
C2	0.0329 (10)	0.0361 (10)	0.0346 (11)	0.0016 (8)	0.0043 (8)	−0.0048 (8)
C3	0.0474 (12)	0.0436 (12)	0.0319 (11)	−0.0050 (10)	0.0047 (9)	−0.0011 (9)
C4	0.0453 (13)	0.0393 (11)	0.0412 (12)	−0.0031 (10)	−0.0107 (10)	0.0048 (9)
C5	0.0357 (12)	0.0435 (12)	0.0560 (14)	0.0076 (9)	−0.0039 (10)	−0.0027 (11)
C6	0.0335 (11)	0.0433 (11)	0.0422 (12)	0.0017 (9)	0.0051 (9)	−0.0029 (9)
C7	0.0325 (11)	0.0382 (10)	0.0343 (11)	0.0002 (8)	0.0067 (8)	−0.0038 (9)
C8	0.0357 (11)	0.0378 (11)	0.0348 (11)	−0.0091 (9)	0.0032 (8)	−0.0022 (8)
C9	0.0550 (15)	0.0684 (16)	0.0587 (16)	0.0019 (13)	0.0281 (12)	0.0064 (13)

### Geometric parameters (Å, °)

**Table:**

Cl1—C2	1.735 (2)	C1—C7	1.455 (3)
Cl2—C4	1.738 (2)	C2—C3	1.378 (3)
N1—C7	1.279 (2)	C3—C4	1.370 (3)
N1—N2	1.373 (2)	C3—H3A	0.9300
N2—C8	1.356 (2)	C4—C5	1.376 (3)
N2—H2	0.898 (10)	C5—C6	1.373 (3)
N3—C8	1.320 (3)	C5—H5	0.9300
N3—C9	1.452 (3)	C6—H6	0.9300
N3—H3	0.893 (10)	C7—H7	0.9300
S1—C8	1.690 (2)	C9—H9A	0.9600
C1—C6	1.393 (3)	C9—H9B	0.9600
C1—C2	1.397 (3)	C9—H9C	0.9600
			
C7—N1—N2	115.91 (17)	C6—C5—C4	119.05 (19)
C8—N2—N1	119.51 (17)	C6—C5—H5	120.5
C8—N2—H2	120.7 (17)	C4—C5—H5	120.5
N1—N2—H2	119.8 (17)	C5—C6—C1	122.07 (19)
C8—N3—C9	123.97 (19)	C5—C6—H6	119.0
C8—N3—H3	118.4 (18)	C1—C6—H6	119.0
C9—N3—H3	117.3 (18)	N1—C7—C1	119.52 (18)
C6—C1—C2	116.50 (18)	N1—C7—H7	120.2
C6—C1—C7	121.54 (17)	C1—C7—H7	120.2
C2—C1—C7	121.95 (17)	N3—C8—N2	116.47 (18)
C3—C2—C1	122.36 (18)	N3—C8—S1	124.78 (15)
C3—C2—Cl1	117.13 (15)	N2—C8—S1	118.75 (15)
C1—C2—Cl1	120.50 (15)	N3—C9—H9A	109.5
C4—C3—C2	118.59 (19)	N3—C9—H9B	109.5
C4—C3—H3A	120.7	H9A—C9—H9B	109.5
C2—C3—H3A	120.7	N3—C9—H9C	109.5
C3—C4—C5	121.40 (19)	H9A—C9—H9C	109.5
C3—C4—Cl2	119.12 (17)	H9B—C9—H9C	109.5
C5—C4—Cl2	119.47 (17)		

### Hydrogen-bond geometry (Å, °)

**Table:**

*D*—H···*A*	*D*—H	H···*A*	*D*···*A*	*D*—H···*A*
N2—H2···S1^i^	0.90 (1)	2.54 (1)	3.4169 (18)	167 (2)
N3—H3···S1^ii^	0.89 (1)	2.77 (2)	3.491 (2)	139 (2)

Symmetry codes: (i) −*x*, *y*, −*z*+3/2; (ii) −*x*+1/2, *y*−1/2, −*z*+3/2.
